# From intent to implementation: Factors affecting public involvement in life science research

**DOI:** 10.1371/journal.pone.0250023

**Published:** 2021-04-28

**Authors:** John A. Burns, Sinead Holden, Kora Korzec, Emma R. Dorris

**Affiliations:** 1 Lamont-Doherty Earth Observatory of Columbia University, Palisades, New York, United States of America; 2 American Museum of Natural History, New York, New York, United States of America; 3 eLife Ambassador for Good Practice in Science, Cambridge, United Kingdom; 4 Clinical Research Centre, School of Medicine, University College Dublin, Dublin, Ireland; 5 eLife Sciences Publishing, Cambridge, United Kingdom; 6 School of Medicine, University College Dublin, Dublin, Ireland; University of Rennes 1, FRANCE

## Abstract

Public involvement is key to closing the gap between research production and research use, and the only way to achieving ultimate transparency in science. The majority of life science research is not public-facing, but is funded by the public and impacts communities. We undertook an exploratory survey of researchers within the life sciences to better understand their views and perceived challenges to involving the public in their research. As survey response rate could not be determined, interpretation of the results must be cautious. We had a valid response cohort of *n* = 110 researchers, of whom 90% were primarily laboratory based. Using a mixed methods approach, we demonstrate that a top-down approach is key to motivate progression of life scientists from feeling positive towards public involvement to actually engaging in it. Researchers who viewed public involvement as beneficial to their research were more likely to have direct experience of doing it. We demonstrate that the systemic flaws in the way life sciences research enterprise is organised, including the promotion system, hyper-competition, and time pressures are major barriers to involving the public in the scientific process. Scientists are also apprehensive of being involuntarily involved in the current politicized climate; misinformation and publicity hype surrounding science nowadays makes them hesitant to share their early and in-progress research. The time required to deliberate study design and relevance, plan and build relationships for sustained involvement, provide and undertake training, and improve communication in the current research environment is often considered nonpragmatic, particularly for early career researchers. In conclusion, a top-down approach involving institutional incentives and infrastructure appears most effective at transitioning researchers from feeling positive towards public involvement to actually implementing it.

## Introduction

There is increasing focus on the translation and implementation of new scientific evidence in real-world settings, in line with the increase in citizen science and public and patient involvement (PPI) initiatives [1]. Across the life sciences, narrowing the gap between research production and research use is a key challenge. There is increasing evidence that public involvement and stakeholder engagement is key to achieving impact and true provision and use of scientific knowledge for the benefit of society [2–8].

**Fig 1:**
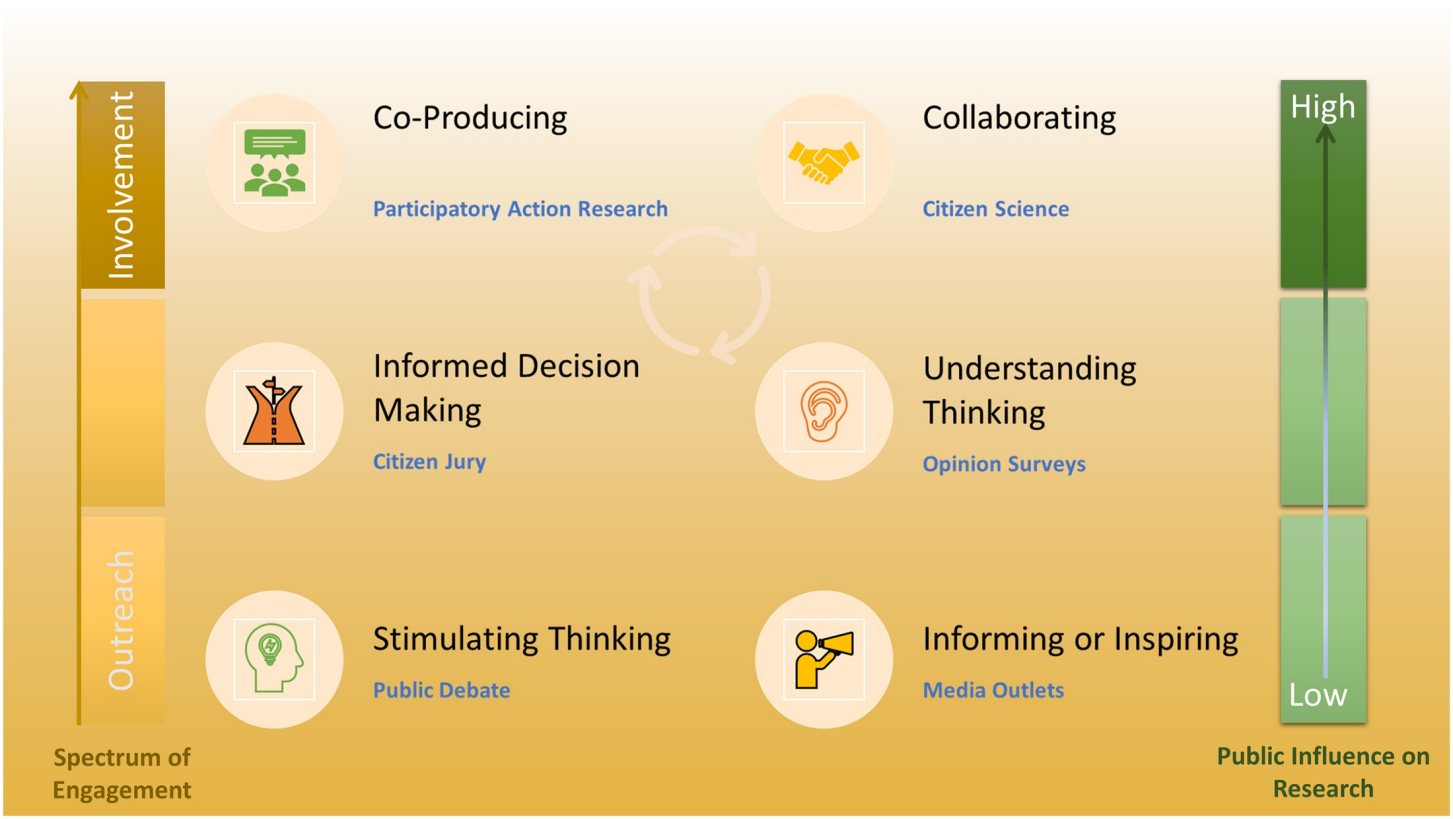
Public involvement within the spectrum of public engagement. Public Engagement is a broad term that includes the multi-faceted and varied ways researchers engage with others outside their field of expertise. Public engagement in research consists of different levels of activities, characterised based on their ability to influence research. These frequently overlap, as successful higher-level engagement is usually built on strong foundational (lower level) engagement.

The field of public engagement has adopted a varied vocabulary where the meanings of specific terms can vary by discipline and by geography. In this study we focus on public involvement in research (Box 1). Public involvement sits in the sphere of public engagement activities (Fig 1). However, public involvement is a collaborative effort between researchers and the public in which the public can have a high level of influence on research. In life science fields not directly related to human health, such as ecological and environmental research, research projects that include members of the public as active participants are often called "citizen science" efforts. Citizen science efforts where members of the public work with professional scientists can be classified into three major categories: contributory, collaborative, and co-created projects [9]. The categories reflect the level of input and participation from members of the public (Fig 1). We use the term public in our context to mean people who are not career researchers in the given field. Although some initiatives may be targeted to reach an interested ‘general public’, targeting the general public as if they were an undifferentiated group of people is rarely successful [10]. The term “public” refers to a targeted audience, be they communities of people based on their interests, passions, or other shared circumstances.

**Box 1. Definitions provided to survey participants prior to answering questions**

**Participation**: public take part in an experiment, trial, or study as a subject. The person who participates is the target of observation by researchers. They are data providers. Examples: participant in a clinical trial.

**Engagement**: a one-way dialogue from researcher to public where the researcher explains/educates/informs the public about their research. Examples: public lectures, lab tours, research demonstrations

**Consultation**: a one-way dialogue from public to researcher where the public’s input on matters affecting them is sought. The public are usually not decision makers. Examples: Surveys or focus groups to inform policy or governance

**Involvement**: two-way dialogue between public and researchers. The active involvement between people who use services, the public and researchers. Public are active collaborators, akin to researchers, clinicians/professionals or managers who are asked for their views and experiences when contributing to research. The public usually have some degree of decision making. Examples: Research advisory groups members, citizen science, public educators or mentors.

Collaborative projects are often designed by scientists but receive input and feedback from participants and legislators to achieve a conservation goal, such as maintaining water quality or protecting habitat for a threatened species [11,12]. Co-created projects occur when members of the public have a say in study design, data collection, data interpretation, and dissemination of results [9]. Co-created projects can help frame concerns of community members as scientific questions and can levy specialized local knowledge and engagement from community members. One such co-created project, "Gardenroots" involved measuring arsenic exposure routes in a community based near a superfund site in Arizona [13]. Involving members of the community in all aspects of the study aided the overall goal of risk communication to the community since members of the community were involved in the actual risk analysis [13]. Successful completion of projects that include a citizen science component is aided by careful and deliberate study design including project goals, scientific questions, plans for sustained participation, training, communication, and desired project outcomes [14].

Public and patient involvement (PPI) in health research is akin to citizen science applied specifically to the health and social care field. The most commonly used PPI definition is “research carried out ‘with’ or ‘by’ members of the public rather than ‘to’, ‘about’ or ‘for’ them” [15]. Both citizen science and PPI are most commonly practiced by public-facing disciplines. Expanding public involvement to laboratory-based and non-public facing disciplines is more challenging for all stakeholders [16–18].

There has been increasing dialogue about public engagement in science. However, there is a risk of public engagement practices being siloed; never quite embedding broadly in fundamental practices, assumptions and research cultures [19]. If publicly engaged science is to have purchase, it must happen even in non-traditional disciplines as well as those scientific communities where the benefits are well-defined and direct [20]. Public engagement with science encompasses the spectrum of intentional, meaningful interactions that provide opportunities for mutual learning between scientists and members of the public. Whereas scientific outreach to the public is common in non-public facing disciplines (Fig 1); public involvement in science (Box 1), which entails collaborations between professional scientists and members of the public who are directly involved in a scientific research project, is far less common [21,22]. The provision of information (outreach) is a necessary aspect of publicly engaged research but is often emphasized at the expense of listening to, acknowledging and incorporating the interests of the public [23].

To embed public involvement practices into non-public facing disciplines will require support and cultivation by researchers themselves. In this study we use a survey of international researchers within the life sciences as a method of understanding the attitudes and challenges of public involvement and to identify the factors that promote public involvement in life science research.

## Materials and methods

An ethics exemption was obtained from the Institutional Review Board of UCD Dublin. Participation in the survey was voluntary, no incentives were offered. This is an exploratory study that was not a priori registered. Informed consent was sought from all participants. Participants were informed of survey anonymity, contact details for the investigators and the purpose of the survey in advance of consenting. No IP addresses were collected. Participants who refused consent, were under the age of 18, or were not life scientists were removed from the dataset.

### Survey development

The survey was developed as per Kelley et al (2003 [24]). Google forms was used as the survey platform. Only nominated members for the project team had access to response data. The usability of the questionnaire was tested by native (*n* = 3) and non-native (*n* = 3) English speaking life scientists. An open format survey was used, with participants self-declaring if they were life scientists. The survey consisted of seven sections:1. About the survey; 2. Consent (*n* = 3 questions); 3. Research Details (*n* = 4 questions); 4. Researcher Demographics (*n* = 6 questions); 5. Public Involvement Awareness and Experience (*n* = 10 questions); 6. Views on Public Involvement (*n* = 11 questions); and 7. Thanks and Follow-up. The survey was presented as one section per page. Sections 2–5 contained nominal questions whereas section five contained both nominal and unrestricted textbox questions. Only the questions on consent were mandatory. A responder progress bar was visible whilst completing the survey. Questions were not randomised. A single question on the age that parents finished education was used as a proxy for researcher socio-economic status (SES) during upbringing [25]. The survey was only available in the English language. The survey can be found in S1 Appendix.

### Sampling and survey administration

Convenience sampling was used via electronic distribution of survey to members of the international working group of life scientists, the eLife Ambassadors for Good Practice in science (*n* = 206). Advertising occurred via a private online cloud-based team collaboration hub and via email, with a request for them to also share it within their scientific communities, societies within the life sciences, and through internal institutional communications. The survey was also advertised via a blog on the National Co-ordinating Centre For Public Engagement (UK) [18]. Data was collected between August-December 2018.

### Quantitative analysis

Log files were checked for duplicate entries. Data was analysed in IBM SPSS v24. Data was analysed via binary regression analysis with public involvement experience (PIE) as the binary (yes/no) dependent variable. ‘No’PIE was used as the reference category.

#### Variable identification

To determine the predictor variables in the model, a binary regression analysis was run with PIE as the binary dependent variable and demographic variables as covariates. Any variables with p<0.1 was carried forward in block 1 to the final model. The demographic variables were as follows: sex (categories: female/male/prefer not to say); age (in years); disability (coded as dichotomous yes/no); ethnic background (coded into 5 categories: 1. Asian (including Asian American & Asian (all other backgrounds), Asian European had a null response; 2. Black (including Black American, the categories Black African, Black (Arab) & Black (Any other background) were excluded as they had a null response); 3. Latino (including Latino American and Latino (Any Other Background); 4. White (including White European, White American, White Arab, White (Any other background); and 5. Other. Nationality, a free test answer, was coded by continent as dichotomous yes/no variables for European, North American, South American, Asian or Oceania. Those who answered countries spanning multiple continents (such as Turkey) were included in both. Africa was excluded as there were no answers from this continent. Socio-economic background (categories: up to age 15, 15–18, 18–23, >23). All covariates except for age were categorical.

A binary regression was run with PIE as the binary dependent variable and research-associated variables as covariates. Any variables with p<0.1 was carried forward in block 2 to the final model. Where do you conduct your primary research was recoded into dichotomous yes/no variables for lab (all lab types), field (all field types), community (any community including national/international) and government (including government facilities and public institutions (museums)). The region researchers are based in was coded into dichotomous variables by continent (Europe, North America, South America, Asian and Oceania). Career stage (categories: 1. Graduate & postgraduate, 2. Postdoctoral and research fellow, 3. Principal Investigator). Respondents could select up to four primary research disciplines. To capture this data for regression, categories were combined into larger groups (1. Cell & Molecular Biology including biochemistry, molecular biology, structural biology, biotechnology, cell biology, nanobiology; 2. Genetics including genetics and genomics, chromosomes and gene expression; 3. Neuroscience; 4. Biomedical and Health-associated including cancer biology, epidemiology and global health, human biology and medicine, immunology, infectious diseases, regenerative medicine, anatomy and physiology; 5. Ecology and conservation biology; 6. Botany and plant science; 7. Developmental and evolutionary science and 8. Computational and Theoretical Biology). These groups were coded dichotomously as yes/no. The final variable included was a dichotomous yes/no response to the question “Have you applied for funding or ethics where there was a specific question on public involvement in research?”.

#### Final model

Data was analysed via logistic regression with public involvement experience (PIE) as the binary (yes/no) dependent variable. ‘No’PIE was used as the reference category. Demographic variables age and disability status (categorical) were included as covariates in block 1. Research associated variables community- based research, government-based research and “Have you applied for funding or ethics where there was a specific question on public involvement in research?” were included as categorical variables in block 2.

### Qualitative analysis

Free text answers underwent qualitative textual analysis. Data were analysed using the pragmatist approach of inductive thematic analysis [26] combined with frequency analysis of themes, and managed using excel [27]. Two researchers (KK; ED, both of whom have experience in open science and qualitative analyses) analysed data independently, with a third independent researcher nominated to mediate potential discrepancies. We used the standard 6-stage analysis: 1. Becoming familiar with the data. 2. Generating initial codes: All relevant phrases, sentences, or text units that related to the research question were extracted into a data display table and given a code 3. Themes: Broad overarching themes were established based on code similarities. 4. Reviewing themes: independently derived themes were reviewed for internal homogeneity and external heterogeneity. 5. Defining and naming themes: the refined themes were discussed and the most apt name given to each identified theme. A quote from the data that captured a distinct aspect of each theme was identified. 6. Producing the report: A detailed account of each theme was composed. Stages 1–3 were performed independently by each researcher. Stages 4–6 were performed as a research team.

## Results

There were 122 respondents, of which 117 individuals self-reported as life scientists. Further five respondents did not consent to the use of their data, and two were under the age of 18, resulting in a final dataset of *n* = 110 respondents. There was a high completeness rate of the survey. Sections on consent, research details, and public involvement awareness and experience had 100% completeness rates. Research demographics had completeness rates ranging from 93.6%-99.1%. The closed questions (*n* = 4) in the Views on Public Involvement section had 100% completion whereas the free text question had a completeness rate between 66.4%-75.6%. The dataset can be found at 10.6084/m9.figshare.12249671. Of the 110 respondents, *n* = 35 (31.8%) had public involvement experience and *n* = 75 did not (68.2%).

### Research demographics

In the dataset of 110 researchers, respondents were overwhelmingly laboratory-based at a university or research institute (*n* = 100 (90.9%); Table 1). Respondents were based in research locations across five continents with no respondents from research institutes in Africa. Most respondents were based in North America or Europe (*n* = 47 (42.8% of all responses) and *n* = 34 (30.9%) respectively) followed by Central and South America (*n* = 16, 14.4%) and Oceania (*n* = 4, 3.6%).

**Table 1:** Response frequencies.

	Frequency		Percent
*Life Science Discipline*			
Cell & Molecular Biology	40		36.4
Genetics	16		14.5
Neuroscience	30		27.3
Biomedical & Health	39		35.5
Ecology & Conservation	5		4.5
Botany & Plant Science	7		6.4
Developmental & Evolutionary	17		15.5
Computational & Theoretical	6		5.5
*Research Setting*			
Laboratory	100		90.9
Community	9		8.2
Field	6		5.5
Government/Public (eg Museum)	9		8.2
*Career Stage*			
Graduate/Postgraduate	33		30.0
Postdoctoral/Fellow	45		40.9
Principal Investigator (PI)	31		28.8
*Public Involvement*			
Would you like to involve the public in your research?	***No***	5	4.6
	***Maybe***	47	42.7
	***Yes***	58	52.7
As far as you know, does your research institute provide resources (excluding funding) for the involvement of the public in research?	***No***	39	35.5
	***Maybe***	16	14.6
	***Yes***	55	50.0
As far as you know, does your research institute encourage the involvement of the public in research?	***No***	25	22.7
	***Yes***	85	77.3
Have you completed any training to facilitate the involvement of the public in your research?	***No***	87	79.1
	***Yes***	23	20.9
Are you aware of online resources to help facilitate public involvement in research?	***No***	75	68.2
	***Yes***	35	31.8
Have you used any online resources for the involvement of the public in research?	***No***	89	80.9
	***Yes***	21	19.1
Have you been offered training to facilitate the involvement of public in your research?	***No***	78	70.9
	***Yes***	32	29.1
Do you think public involvement in research is beneficial to the general public?	***No***	1	0.9
	***Maybe***	12	10.9
	***Yes***	97	88.2
Do you think public involvement in research is beneficial to research?	***No***	1	0.9
	***Yes***	35	31.8
	***Maybe***	74	51.9
Do you think public involvement in research is beneficial to you as a researcher?	***No***	10	9.1
	***Maybe***	43	39.1
	***Yes***	57	51.8
Do you think you think some level of public involvement should be a requisite/part of the terms of funding for all research projects? [*Publicly funded projects only]	***No***	44	40.0
	***Maybe***	9	8.2
	***Yes***	16	14.6
	Maybe*	24*	21.8
	Yes*	17*	15.5
Have you involved the public in your research?	***No***	75	68.2
	***Yes***	35	31.8

### Characteristics associated with public involvement experience

Five variables were brought forward for inclusion in the final model: age, disability, community- based research setting, government-based research setting and “Have you applied for funding or ethics where there was a specific question on public involvement in research?”. When these variables were included in the final model (Model Chi Squared statistic 30.21, *p*<0.001), only answering “Yes” to applying for funding or ethics where there was a specific question on public involvement in research remained associated with PIE (p<0.001, OR 6.36 (95% CI 2.31–17.51), Table 2).

**Table 2:** Binary logistic regression model for characteristics associated with PIE.

	Value (Log Odds Units)	Standard Error	Wald chi-square value	p-value (2-tailed)	Odds Ratio (OR)	95% CI for OR	
						Lower	Upper
Age	-0.06	0.03	3.72	0.054	0.945	0.893	1.001
Having a Disability	1.23	0.66	3.48	0.062	3.418	0.939	12.437
Community-based research	1.53	0.92	2.79	0.095	4.661	0.767	28.339
Government-based research	1.03	0.81	1.61	0.204	2.804	0.571	13.770
Have you applied for funding or ethics where there was a specific question on public involvement in research? (’Yes’)	1.85	0.52	12.80	0.000	6.357	2.308	17.508
Constant	-1.74	1.66	1.10	0.293	0.175		

### Researcher-perceived barriers to public involvement

As per Maccarthy et al (2018), challenges surrounding public involvement were asked in the context of institutional barriers, personal worries and research concerns [17]. There were 81 responses to the question on institutional barriers [In your view, what are the institutional barriers to including public involvement in your research?]. The well documented challenges of regulations, resources and funding were major themes emerging from the data [7,17,28,29]. The other major theme was that of career benefit.

“Not valued as academic currency for TT*/jobs.”

- Postdoctoral researcher in cell biology, based in North America (*TT: Tenure Track)

We had 79 responses to the question on personal barriers to involvement, of which *n* = 9 (11.4%) indicated they did not have any personal barriers. Within the remaining responses to the question [In your view, what are the personal barriers (worries) that would prevent you including public involvement in your research?] two main, and highly related, themes emerged: fear of misrepresentation and personal communication skills.

When asked expressly about the concerns of public involvement to their research [In your view, what are the research concerns that would prevent you including public involvement in your research?]; of the 70 respondents, 15 (21.4%) said they had no concerns about its impact on their research. Of the remaining respondents: misrepresentation, workload, effect on research focus and relevance of public involvement for their research emerged as concerns with approximately equal frequency.

“*The main barrier is time—being involved in public outreach takes precious time away from research activities, getting grants and publications*.”

- Principal Investigator, cancer and cell biology, Europe.

Time is a precious resource in the life sciences and burnout is common [30–33]. Even when researchers acknowledged the potential benefit of public involvement to their research, there was concern that there were not sufficient resources in place such that the benefit to their research would not outweigh the cost in terms of time.

## Discussion

Science can be a public good and a social enterprise. In addition to inordinate technological development, the rapid growth in life sciences research and knowledge has occurred due to a long-standing public investment in life science research [34–37]. The fact that much research is not public-facing does not mean that it is not publicly relevant. In order to continue sustained investment into life science research, we must build more comprehensive, interactive and mutually beneficial opportunities for dialogue and exchange with our largest funders- the public [38]. Openness in science does not solely relate to article processing fees [39]. Responsibility and openness in science also relates to increasing the accessibility and use of scientific knowledge. To this end, responsible science should include appropriate dialogue with the community upon which it has an impact; or which it may be perceived to impact [20,40].

Here we demonstrate that a top-down approach encourages public involvement in life sciences research. Our analyses found that the strongest correlate to actual experience of public involvement in research was applying to institutional bodies that specifically asked researchers about their public involvement activities. Funders and institutions providing the mandate and resources to support public involvement is paramount to foster public involvement. Simply feeling positively towards involving the public in research was not sufficient for researchers to implement it. Researchers who considered public involvement to be beneficial to the researcher themselves or to their research, rather than just to the general public, were more likely to engage in it. This suggests that provision of greater context or immersive experience of public involvement in the life sciences would allow researchers to understand and conceptualise the potential benefit of public involvement to their own research.

Previous findings from public engagement and public communications have demonstrated the likelihood of having involved the pubic in research increased with age. Although age was a significant factor in the initial model where only demographic variables were included, age was not significant in our final model that also included research-associated variables (*p* = 0.054) [41,42]. Our findings therefore diverge with the literature demonstrating that both scientific engagement and active dissemination increase with age and experience [41,42]. It should be noted, however, that those studies focused predominantly on outreach activities, and were centred on researchers in a single university [42] or a single, albeit large, national research organisation [41]. This, in combination with the fact that those studies were conducted pre the social media age, may explain this divergence with our findings.

Our qualitative analysis identified that the career pathway, including funding pressures and the promotion system of research institutions has a key role to play in the pragmatic ability of early-career researchers to truly consider public involvement. This highlights that simply providing training and resources for public involvement, without implementing changes in the science career pathways, may have limited benefit at an early career stage [43].

“Fear of being misrepresented or being "less serious"”

- Postgraduate conservation biologist, South America

Involving the public leaves the researcher and their research vulnerable to misrepresentation and being negatively viewed by your peers and colleagues. This finding is consistent with Poliakoff and Webb [42] who found that colleagues participating had a positive influence on a researcher’s intent to engage the public. However, other studies of scientists’ willingness to engage found descriptive norms to be relatively limited predictors of willingness to engage[44,45].

Public involvement is demanding both in terms of time and resources. The academic career structure and the increasingly high bar to earn job stability or tenure as an academic researcher is a major barrier to public involvement [46,47].

“The extremely competitive science system preclude me to really consider it”

- Research Fellow in biochemistry, based in Europe.

Institutions need to consider not only the funding and resources available for public involvement, but the environment necessary to promote it. The continued focus on academic productivity, at the expense of academic activities that grow or improve research, will continue to restrict researchers in their innovative potential and growth as pragmatism and limited time and energy necessitates focus on tenure track and promotion variables. The attitude of “waiting for tenure to do what you actually love” has become pervasive in the life sciences. However, many excellent and innovative scientists are lost to science during the long road to tenure [36,48,49]. Engaging stakeholders is beneficial to science and to the translation and use of scientific knowledge [2,6,50] and institutions need to consider the life sciences system as a whole in their efforts to encourage stakeholder engagement and knowledge transfer [36].

Researchers who had involved the public in their research were more knowledgeable about the local and electronic resources available to them and more likely to have engaged in training for public involvement. These resources seem helpful downstream, once public involvement has been initiated. Science communication is a critical skill for public involvement. Without some grasp of the relevant science, the public cannot be expected to make informed decisions about research [51]. In the absence of a pre-existing relationship, how can a researcher understand the public’s information needs, and make it accessible in a format useful for public involvement? Can we expect laboratory-based life scientists to also be skilled in bridging science and decision making? There are frameworks available to assist in science communication but the evidence-base for their effectiveness in the life sciences is understudied [52–54].

“Once misinformation has been disseminated, it seems very difficult to rectify in the current information sharing climate”

- Postdoc in Neuroscience, based in Europe

Science has become increasingly politicised in the public sphere [55]. Political orientations and ideologies can shape public trust in science [55,56]. Fear of misrepresentation of their research, either intentional or otherwise, was a major theme identified in our data.

“There is always a fear that your words might be taken out of context to push an agenda that you don’t agree with. Also, putting yourself out there may make you vulnerable to verbal or even physical attacks on you”

- Principal Investigator in Biochemistry, based in Europe

Complicating the issue is the reach of misinformation. Once misinformation has entered the public sphere, rebuttal with corrections to the misinformation tend to be muted compared with the propagation of the misinformation, leading to false and misrepresented information remaining within the pool of common knowledge [57]. Our data shows this has made researchers wary of opening their in-progress research to the public, even when they recognise there are potential benefits to their research of doing so.

Politicized environments often induce suspicions about science communicators’ true motives or expertise and questions may arise as to whether a scientist can really be trusted [58]. There is a fear among scientists about the potential personal backlash stemming from public involvement. This links to the theme of career benefit.

Researcher vulnerability is an important issue often overlooked in public involvement. For institutions wishing to encourage increased public involvement they may need to consider what protections are in place for researchers exposing their early stage and in-development research to the public sphere. The time required to deliberate study design and relevance, plan and relationship build for sustained involvement, provide and undertake training, and improve communication in the current research environment is not considered feasible within their research and/or institutional environment for many researchers and is an area in need of further research into the best ways to overcome these challenges. Misrepresentation (intentional or otherwise) coupled with the ecosystem of rapid spread of false information on public platforms, and the fear thereof, is a complex issue [59]. It is made more complex in the context of non-public interfacing research, which does not have a high level of public understanding in the first place [60–62]. To truly integrate public involvement in a mutually beneficial way in non-public-facing disciplines requires deeper understanding and improved holistic support for this already over-burdened researcher demographic [36].

This study highlights the challenges faced in compelling researchers, particularly early-career laboratory science researchers, to engage directly with the public by involving them in their research projects. Despite the challenges highlighted here, citizen science efforts have been growing in terms of number of projects [63], outcomes in terms of publications [63], and the number or resources available to researchers and members of the public alike including open source, peer reviewed journals on citizen science and public and patient involvement [64–67]. Our results emphasize the need for integration and incentivization of the use of these resources by institutions and funding agencies to maximize researcher’s interests in public involvement [43]. Effective change towards a more open and inclusive model of life sciences will require more than just training and funding. Policy makers and institutions can greatly influence the implementation of public involvement in research through the working conditions and environment they promote.

## Limitations

The sample size of 110 researchers is small, limiting the capacity to conduct more in-depth statistical associations. The high rate of consensus on the beneficial nature of public involvement indicates selection bias in the respondents toward those who view public involvement positively. The survey was only available in English, limiting the potential respondents to those proficient in written English. We are unable to estimate a response rate due to the method of survey advertising. As such, caution must be used in interpretation of the results.

## Conclusions

Fundamentally, a researcher’s decision to involve the public will be decided by whether they believe it has sufficient potential value to both themselves and their research as to warrant the investment in terms of time, energy and potential or perceived career consequences. Policy makers and institutions can greatly influence this decision by creating an environment supportive of responsible research practices, including public involvement. Creating this environment in many cases will require a paradigm shift if public involvement is to become more than a marginal curiosity or tokenistic effort in laboratory-based research [23].

## Supporting information

S1 Table Sex frequency data. (DOCX)

S2 Table Age frequency data. (DOCX)

S3 Table Nationality frequency data. (DOCX)

S4 Table Ethnic/Cultural background frequency data. (DOCX)

S5 Table Socio-Economic frequency data. (DOCX)

S6 Table Disability frequency data. (DOCX)

S7 Table Respondents who have been asked a specific question on public involvement on an ethics or funding application frequency data. (DOCX)

S1 Appendix Public involvement in research questionnaire. (XLSX)
