# Supplementary material for: From intent to implementation: Factors affecting public involvement in life science research
Source: PLoS One. 2021 Apr 28;16(4):e0250023. doi: 10.1371/journal.pone.0250023 (PMC8081191; doi:10.1371/journal.pone.0250023)
Supplement: S1 Table — (DOCX) [file pone.0250023.s001.docx]

| **Table S1:** Sex frequency data  **What sex are you?** | | | | |
| --- | --- | --- | --- | --- |
|  | Frequency | Percent | Valid Percent | Cumulative Percent |
| Male | 61 | 55.5 | 55.5 | 55.5 |
| Female | 47 | 42.7 | 42.7 | 98.2 |
| Prefer not to say | 2 | 1.8 | 1.8 | 100.0 |
| Total | 110 | 100.0 | 100.0 |  |
