# Supplementary material for: From intent to implementation: Factors affecting public involvement in life science research
Source: PLoS One. 2021 Apr 28;16(4):e0250023. doi: 10.1371/journal.pone.0250023 (PMC8081191; doi:10.1371/journal.pone.0250023)
Supplement: S2 Table — (DOCX) [file pone.0250023.s002.docx]

**Table S2:** Age frequency data

| **What age are you?** | | | | |
| --- | --- | --- | --- | --- |
|  | Frequency | Percent | Valid Percent | Cumulative Percent |
| 32 | 12 | 10.9 | 11.4 | 11.4 |
| 28 | 7 | 6.4 | 6.7 | 18.1 |
| 35 | 7 | 6.4 | 6.7 | 24.8 |
| 23 | 6 | 5.5 | 5.7 | 30.5 |
| 25 | 6 | 5.5 | 5.7 | 36.2 |
| 39 | 6 | 5.5 | 5.7 | 41.9 |
| 30 | 5 | 4.5 | 4.8 | 46.7 |
| 34 | 5 | 4.5 | 4.8 | 51.4 |
| 26 | 4 | 3.6 | 3.8 | 55.2 |
| 36 | 4 | 3.6 | 3.8 | 59.0 |
| 38 | 4 | 3.6 | 3.8 | 62.9 |
| 40 | 4 | 3.6 | 3.8 | 66.7 |
| 44 | 4 | 3.6 | 3.8 | 70.5 |
| 29 | 3 | 2.7 | 2.9 | 73.3 |
| 33 | 3 | 2.7 | 2.9 | 76.2 |
| 37 | 3 | 2.7 | 2.9 | 79.0 |
| 42 | 3 | 2.7 | 2.9 | 81.9 |
| 24 | 2 | 1.8 | 1.9 | 83.8 |
| 27 | 2 | 1.8 | 1.9 | 85.7 |
| 41 | 2 | 1.8 | 1.9 | 87.6 |
| 53 | 2 | 1.8 | 1.9 | 89.5 |
| 61 | 2 | 1.8 | 1.9 | 91.4 |
| 21 | 1 | 0.9 | 1.0 | 92.4 |
| 22 | 1 | 0.9 | 1.0 | 93.3 |
| 43 | 1 | 0.9 | 1.0 | 94.3 |
| 45 | 1 | 0.9 | 1.0 | 95.2 |
| 48 | 1 | 0.9 | 1.0 | 96.2 |
| 49 | 1 | 0.9 | 1.0 | 97.1 |
| 57 | 1 | 0.9 | 1.0 | 98.1 |
| 62 | 1 | 0.9 | 1.0 | 99.0 |
| 63 | 1 | 0.9 | 1.0 | 100.0 |
| Total | 105 | 95.5 | 100.0 |  |
| Missing | 5 | 4.5 |  |  |
|  | 110 | 100.0 |  |  |
