# Supplementary material for: From intent to implementation: Factors affecting public involvement in life science research
Source: PLoS One. 2021 Apr 28;16(4):e0250023. doi: 10.1371/journal.pone.0250023 (PMC8081191; doi:10.1371/journal.pone.0250023)
Supplement: S3 Table — (DOCX) [file pone.0250023.s003.docx]

**Table S3:** Nationality frequency data

| **What nationality are you?** | | | | |
| --- | --- | --- | --- | --- |
|  | Frequency | Percent | Valid Percent | Cumulative Percent |
| USA | 35 | 31.8 | 32.4 | 32.4 |
| Chile | 10 | 9.1 | 9.3 | 41.7 |
| India | 10 | 9.1 | 9.3 | 50.9 |
| Ireland | 8 | 7.3 | 7.4 | 58.3 |
| UK | 4 | 3.6 | 3.7 | 62.0 |
| Canada | 4 | 3.6 | 3.7 | 65.7 |
| France | 4 | 3.6 | 3.7 | 69.4 |
| The Netherlands | 3 | 2.7 | 2.8 | 72.2 |
| Germany | 3 | 2.7 | 2.8 | 75.0 |
| New Zealand | 3 | 2.7 | 2.8 | 77.8 |
| Australia | 2 | 1.8 | 1.9 | 79.6 |
| Brazil | 2 | 1.8 | 1.9 | 81.5 |
| Iran | 2 | 1.8 | 1.9 | 83.3 |
| Italian | 2 | 1.8 | 1.9 | 85.2 |
| Luxembourg | 2 | 1.8 | 1.9 | 87.0 |
| Singapore | 2 | 1.8 | 1.9 | 88.9 |
| Argentina | 1 | 0.9 | 0.9 | 89.8 |
| Austria | 1 | 0.9 | 0.9 | 90.7 |
| Belgium | 1 | 0.9 | 0.9 | 91.7 |
| Columbia | 1 | 0.9 | 0.9 | 92.6 |
| Czech Republic | 1 | 0.9 | 0.9 | 93.5 |
| Mexico | 1 | 0.9 | 0.9 | 94.4 |
| Poland | 1 | 0.9 | 0.9 | 95.4 |
| Spain | 1 | 0.9 | 0.9 | 96.3 |
| Switzerland | 1 | 0.9 | 0.9 | 97.2 |
| Taiwan | 1 | 0.9 | 0.9 | 98.1 |
| Turkey | 1 | 0.9 | 0.9 | 99.1 |
| Vietnam | 1 | 0.9 | 0.9 | 100.0 |
| Total | 108 | 98.2 | 100.0 |  |
