# Supplementary material for: From intent to implementation: Factors affecting public involvement in life science research
Source: PLoS One. 2021 Apr 28;16(4):e0250023. doi: 10.1371/journal.pone.0250023 (PMC8081191; doi:10.1371/journal.pone.0250023)
Supplement: S4 Table — (DOCX) [file pone.0250023.s004.docx]

**Table S4:** Ethnic/Cultural Background frequency data

| **What is your Ethnic/Cultural Background?** | | | | |
| --- | --- | --- | --- | --- |
|  | Frequency | Percent | Valid Percent | Cumulative Percent |
| White European | 42 | 38.2 | 38.5 | 38.5 |
| White American | 18 | 16.4 | 16.5 | 55.0 |
| Asian (Any other background) | 15 | 13.6 | 13.8 | 68.8 |
| Latino American | 12 | 10.9 | 11.0 | 79.8 |
| White (Any other background) | 6 | 5.5 | 5.5 | 85.3 |
| Other (Including mixed backgrounds) | 6 | 5.5 | 5.5 | 90.8 |
| Asian American | 4 | 3.6 | 3.7 | 94.5 |
| Black American | 3 | 2.7 | 2.8 | 97.2 |
| Latino (Any Other Background) | 2 | 1.8 | 1.8 | 99.1 |
| White (Arab) | 1 | 0.9 | 0.9 | 100.0 |
| Total | 109 | 99.1 | 100.0 |  |
| Missing | 1 | 0.9 |  |  |
|  | 110 | 100.0 |  |  |
