# Supplementary material for: From intent to implementation: Factors affecting public involvement in life science research
Source: PLoS One. 2021 Apr 28;16(4):e0250023. doi: 10.1371/journal.pone.0250023 (PMC8081191; doi:10.1371/journal.pone.0250023)
Supplement: S5 Table — (DOCX) [file pone.0250023.s005.docx]

**Table S5:** Socio-Economic frequency data

| **Socio-economic background: At what age did your parent/guardian leave continuous full-time education?** | | | | |
| --- | --- | --- | --- | --- |
|  | Frequency | Percent | Valid Percent | Cumulative Percent |
| over 23 | 44 | 40.0 | 41.1 | 41.1 |
| 18-23 | 41 | 37.3 | 38.3 | 79.4 |
| 15-18 | 16 | 14.5 | 15.0 | 94.4 |
| up to 15 | 6 | 5.5 | 5.6 | 100.0 |
| Total | 107 | 97.3 | 100.0 |  |
| Missing | 3 | 2.7 |  |  |
|  | 110 | 100.0 |  |  |
