# Supplementary material for: From intent to implementation: Factors affecting public involvement in life science research
Source: PLoS One. 2021 Apr 28;16(4):e0250023. doi: 10.1371/journal.pone.0250023 (PMC8081191; doi:10.1371/journal.pone.0250023)
Supplement: S6 Table — (DOCX) [file pone.0250023.s006.docx]

**Table S6:** Disability frequency data

| **Do you have a disability?** | | | | |
| --- | --- | --- | --- | --- |
|  | Frequency | Percent | Valid Percent | Cumulative Percent |
| No | 90 | 81.8 | 85.7 | 85.7 |
| Vision | 5 | 4.5 | 4.8 | 90.5 |
| Autism Spectrum | 3 | 2.7 | 2.9 | 93.3 |
| Hearing | 3 | 2.7 | 2.9 | 96.2 |
| Mental Health | 3 | 2.7 | 2.9 | 99.0 |
| Intellectual | 1 | 0.9 | 1.0 | 100.0 |
| Total | 105 | 95.5 | 100.0 |  |
| System | 5 | 4.5 |  |  |
|  | 110 | 100.0 |  |  |
