# Supplementary material for: From intent to implementation: Factors affecting public involvement in life science research
Source: PLoS One. 2021 Apr 28;16(4):e0250023. doi: 10.1371/journal.pone.0250023 (PMC8081191; doi:10.1371/journal.pone.0250023)
Supplement: S7 Table — (DOCX) [file pone.0250023.s007.docx]

**Table S7:** Respondents who have been asked a specific question on public involvement on an ethics or funding application frequency data

| **Have you applied for funding or ethics where there was a specific question on public involvement in research?** | | | | |
| --- | --- | --- | --- | --- |
|  | Frequency | Percent | Valid Percent | Cumulative Percent |
| No | 75 | 68.2 | 68.2 | 68.2 |
| Yes | 35 | 31.8 | 31.8 | 100.0 |
| Total | 110 | 100.0 | 100.0 |  |
